# Firefly Luciferase and Rluc8 Exhibit Differential Sensitivity to Oxidative Stress in Apoptotic Cells

**DOI:** 10.1371/journal.pone.0020073

**Published:** 2011-05-13

**Authors:** Julie Czupryna, Andrew Tsourkas

**Affiliations:** Department of Bioengineering, School of Engineering and Applied Science, University of Pennsylvania, Philadelphia, Pennsylvania, United States of America; University of Hong Kong, Hong Kong

## Abstract

Over the past decade, firefly Luciferase (fLuc) has been used in a wide range of biological assays, providing insight into gene regulation, protein-protein interactions, cell proliferation, and cell migration. However, it has also been well established that fLuc activity can be highly sensitive to its surrounding environment. In this study, we found that when various cancer cell lines (HeLa, MCF-7, and 293T) stably expressing fLuc were treated with staurosporine (STS), there was a rapid loss in bioluminescence. In contrast, a stable variant of Renilla luciferase (RLuc), RLuc8, exhibited significantly prolonged functionality under the same conditions. To identify the specific underlying mechanism(s) responsible for the disparate sensitivity of RLuc8 and fLuc to cellular stress, we conducted a series of inhibition studies that targeted known intracellular protein degradation/modification pathways associated with cell death. Interestingly, these studies suggested that reactive oxygen species, particularly hydrogen peroxide (H_2_O_2_), was responsible for the diminution of fLuc activity. Consistent with these findings, the direct application of H_2_O_2_ to HeLa cells also led to a reduction in fLuc bioluminescence, while H_2_O_2_ scavengers stabilized fLuc activity. Comparatively, RLuc8 was far less sensitive to ROS. These observations suggest that fLuc activity can be substantially altered in studies where ROS levels become elevated and can potentially lead to ambiguous or misleading findings.

## Introduction

Bioluminescence is widely considered an attractive platform for many molecular imaging applications given its sensitivity, cost-effectiveness, simplicity, high-throughput screening potential and ability to garner temporal information in cells and in live animal models. Bioluminescent reporters are regularly used to study the regulation of gene expression by cis- or trans-acting factors, such as gene regulatory elements[1], transcription factors[2], or exogenous regulators[3]. Luciferase enzymes have also been used for tracking cell migration[4] in living subjects and monitoring intracellular molecular interactions[5].

Since bioluminescent assays often involve studying the cellular response to stimulatory and/or death-inducing conditions, luciferase enzymes can often be exposed to harsh intracellular environments. For example, during apoptosis various proteases (e.g. caspases[6], cathepsins[7], calpains[8]) can be activated or released into the cytosol, proteasomal activity can be upregulated as a result of increased protein ubiquitination[9], and reactive oxygen species (ROS) can create a state of oxidative stress[10]. Each of these mechanisms can potentially alter or impair bioluminescent enzyme function and ultimately interfere with the outcome of reporter gene assays.

To avoid the premature loss of bioluminescent activity, various bioluminescent proteins have been engineered to possess increased stability. Some examples include variants of Firefly luciferase (fLuc) that exhibit increased pH stability and thermostability up to 45°C [11], have a 3-fold longer half-life (T_1/2_) *in vivo* compared to wild-type fLuc [12], or take up to 10-fold longer to decay compared to wild-type fLuc [13]. Similarly, a variant of Renilla Luciferase (rLuc), rLuc8, has been engineered that demonstrates a 200-fold higher resistance to serum inactivation and a light output that is 4-fold greater than wild-type rLuc [14]. Given the improved optical properties of these mutants, one or more of them could be a more desirable candidate for molecular imaging studies involving cellular stresses. Consistent with this hypothesis, we found that RLuc8 was significantly more resistant to inactivation in apoptotic cells, compared with fLuc (see Results). Although these findings were not surprising, it remained unclear which intracellular mechanisms were responsible for the rapid loss in fLuc activity. To elucidate the root cause for fLuc inactivation, a series of inhibition studies were conducted that targeted various intracellular protein degradation/modification pathways associated with apoptosis. Surprisingly, these experiments suggested that ROS, particularly hydrogen peroxide (H_2_O_2_), were responsible for the loss in fLuc activity. Notably, RLuc8 was far less sensitive to ROS. These results were confirmed by subjecting cells, expressing fLuc and RLuc8, to various conditions that resulted in a specific increase or decrease in H_2_O_2_ levels. Considering the growing data linking elevated levels of ROS to various pathologies including atherosclerosis, cancer, cystic fibrosis, type-2 diabetes, and Alzheimer's disease, these findings suggest that the use of fLuc could potentially lead to ambiguous or misleading findings when applied to these systems.

## Results

### RLuc8 is more resistant to inactivation in apoptotic cells, compared with fLuc, resulting in an increase in the RLuc8:fLuc bioluminescence ratio

**Figure 1:**
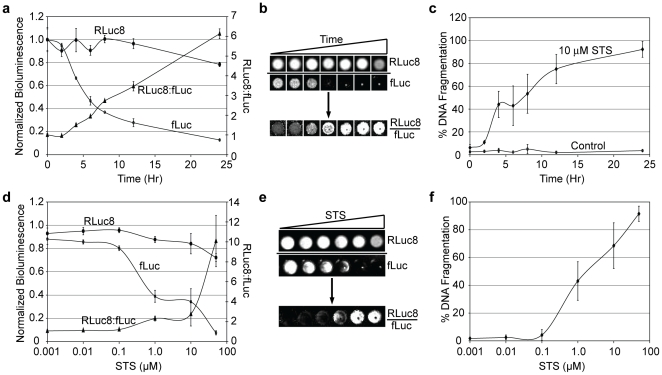
Response of HeLa-fR cells to STS. HeLa-fR cells were treated with 10 µM STS for 24 hours. (a) Bioluminescent measurements of RLuc8 and fLuc were acquired at various times during the course of treatment. All measurements were normalized to values at 0 hours. The RLuc8:fLuc ratio was subsequently calculated for each time point (right axis). (b) A representative bioluminescent image of RLuc8 and fLuc activity in STS-treated cells is shown as well as the calculated ratiometric image, RLuc8:fLuc. (c) A TUNEL assay for DNA fragmentation was also performed at various times during the course of STS treatment. Analogous bioluminescent and DNA fragmentation measurements were performed on RBS – HeLa cells that were treated with a dosage range of STS for 6 hours. (d) Bioluminescent measurements of RLuc8 and fLuc activity in cells treated with various doses of STS. The RLuc8:fLuc ratio is also shown (right axis). (e) Representative bioluminescent images of RLuc8 and fLuc activity in cells treated with various doses of STS. The calculated ratiometric image of RLuc8:fLuc is also shown. (f) A TUNEL assay for DNA fragmentation was also performed on HeLa-fR cells treated with various doses of STS.

When HeLa cells expressing fLuc and RLuc8 (i.e. HeLa-fR) were treated with 10 µM staurosporine (STS), a drug that induces apoptosis[15], the bioluminescent signal from fLuc was significantly reduced over a time period of 24 hours, while the signal from Rluc8 remained comparatively stable (Figure 1a). This differential sensitivity to cellular stress can be represented as an increase in the bioluminescent ratio (Rluc8 activity:fLuc activity), as shown on the secondary axis in Figure 1a. Representative bioluminescent images obtained from HeLa-fR cells under the same conditions are shown in Figure 1b. Little to no increase in the Rluc8:fLuc ratio was observed in cells that were not treated with STS over the same time period (Figure S1a). Further, the Rluc8:fLuc ratio was independent of cell number (Figure S1b). A TUNEL assay confirmed that the STS-treatment was sufficient to induce cell death over the indicated time course (Figure 1c) and a caspase-GLO® 3/7 assay (Promega) confirmed that cell death was caspase-dependent (Figure S2a).

When HeLa-fR cells were treated with increasing doses of STS, the Rluc8:fLuc ratio and extent of cell death also increased in a dose-dependent manner (Figure 1d–f and Figure S2b, respectively). Importantly, these findings were not unique to STS-treated HeLa cells. An increase in the RLuc8:fLuc ratio was also observed when MCF7 (Figure S3a) and 293T/17 (Figure S3d) cells were treated with STS. In both cases, cell death was confirmed by TUNEL assays (Figure S3b and Figure S3e, respectively) and caspase involvement was confirmed using caspase-GLO® 3/7 (Figure S3c and S3f, respectively).

Although it was not surprising that RLuc8 exhibited prolonged bioluminescent activity in apoptotic cells, compared with fLuc, it remained unclear which intracellular mechanism(s) was specifically responsible for the loss in fLuc activity. Since all measurements of fLuc and RLuc8 activity that are shown were acquired using the *in vitro* assay kit, Dual-Glo (Promega), a lack of ATP, Mg^2+^, and oxygen could immediately be ruled out as factors contributing to changes in the Rluc8:fLuc ratio in STS-treated cells. It should be noted that similar bioluminescent measurements were obtained from live and lysed cells, nonetheless. Changes in the Rluc8:fLuc ratio were also observed regardless of whether the coding sequences for fLuc and RLuc8 were interchanged, relative to the intervening internal ribosomal entry site, IRES (Figure S4). Therefore, the presence of an IRES sequence can also be ruled out as a factor contributing to the changes in the RLuc8:fLuc ratio in STS-treated cells.

### Protein levels, but not mRNA levels mimic bioluminescent data

**Figure 2:**
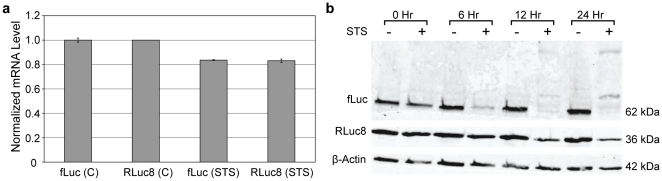
Assessment of mRNA and protein levels in STS-treated HeLa-fR cells. (a) Normalized RLuc8 and fLuc mRNA levels in HeLa-fR cells treated with PBS (control, C) or 10 µM STS (STS) for 24 hours. mRNA levels were determined by qRT-PCR. (b) Western blot of HeLa-fR cells treated with PBS (-) or 10 µM STS (+) over a time course of 24 hours. β-actin is shown as a loading control.

Quantitative RT-PCR and western blot analyses were performed to determine whether changes in RNA or protein levels were responsible for the preferential loss of fLuc activity, compared with RLuc8 activity. Previous studies have shown that oxidative stress can trigger the degradation of both mRNA [16] and proteins [17]. If RNA was degraded, a drastic difference in the relative level of fLuc and Rluc8 mRNA expression would be expected after STS treatment, compared with PBS-treated controls; however, this was not the case. Although slight reductions in fLuc and RLuc8 RNA expression were observed in STS-treated cells, the relative expression remained constant (Figure 2a). This was not entirely surprising considering that fLuc and RLuc8 were expressed as a single bicistronic RNA with an internal ribosomal entry site (IRES). Since a single CMV promoter is used to drive fLuc and RLuc expression, the differential response of fLuc and RLuc8 activity to apoptosis also cannot be attributed to any difference or changes in promoter activity.

In contrast to RNA expression, western blot analysis revealed that fLuc protein levels decreased over the time course of STS treatment, while Rluc8 protein levels remained relatively stable, mirroring the bioluminescent measurements (Figure 2b). Additionally, several higher molecular weight bands were observed after fLuc staining at the later time points, particularly 24 hours, introducing the possibility of protein cross-linking and/or post-translational modification.

### Luciferin and Coelenterazine are not sensitive to intracellular species or enzymes in stressed cells

Although western blotting revealed that there was a differential loss in fLuc protein compared with RLuc8 in STS-treated cells (Figure 2), because fLuc and RLuc8 also react with different substrates (i.e. D-luciferin and coelenterazine, respectively) it remained possible that various intracellular species or enzymes could also exhibit a differential effect on the stability of these substrates. To explore this possibility, wild-type HeLa cells were treated either with STS or PBS for 24 hours. Subsequently, both D-luciferin and coelenterazine (i.e. Dual-Glo) were added to the cells, analogous to the studies above. This was followed by the addition of purified fLuc and RLuc8 proteins. In this approach, the bioluminescent proteins were not exposed to the harsh intracellular environment during STS treatment and therefore would, presumably, not be significantly affected by the intracellular milieu. Bioluminescent measurements confirmed that at least within the time frame of bioluminescence acquisition, intracellular factors did not exhibit any appreciable effect on the fLuc or RLuc8 activity. Therefore, it can be concluded that the intracellular species and enzymes present in apoptotic cells also did not have an appreciable affect on the stability of D-luciferin and coelenterazine (Figure S5).

### H_2_O_2_ is prominently involved in the discrepancy between fLuc and RLuc8 bioluminescence

**Figure 3:**
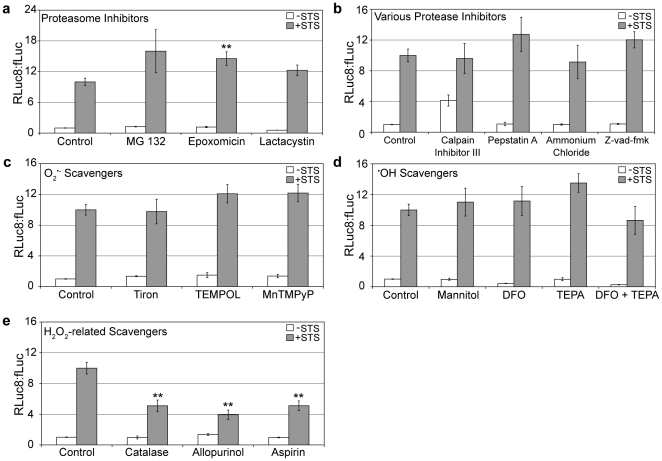
Inhibition studies to determine mechanism responsible for activation of the RBS. HeLa-fR cells were pretreated with (a) proteasome inhibitors, (b) protease inhibitors, (c) O_2_^•-^ scavengers, (d) ^•^OH scavengers, or (e) H_2_O_2_-related scavengers for 1 hour, followed by PBS (white bars) or 10 µM STS for 24 hours (gray bars). The RLuc8:fLuc ratio was calculated and reported. Statistical significance between control (+STS) and individual inhibitors/scavengors (+STS) is indicated with a ** (p<0.01).

To explicate the root cause responsible for the loss in fLuc activity in STS-treated cells and the corresponding increase in the Rluc8:fLuc ratio, systematic inhibition/scavenger studies were performed to individually silence key pathways known to cause protein degradation/modification. Figure S6 outlines some of the key pathways that were investigated, which included proteasomal and lysosomal pathways, apoptotic pathways, and oxidative stress. The various inhibitors/scavengers that were tested and their targets are listed in Table 1. It was expected that certain inhibitors would rescue fLuc activity, resulting in a corresponding decrease in the RLuc8:fLuc ratio in STS-treated HeLa-fR cells. Since the proteasome is a prominent source of intracellular protein degradation, it was naturally one of the first molecular entities we investigated for the inhibition studies. Surprisingly, employment of the proteasome inhibitors MG-132, epoxomicin and lactacystin did not rescue fLuc activity and actually led to an increase in the RLuc8:fLuc ratio (Figure 3a), i.e. fLuc activity was even further reduced relative to RLuc8. The efficacy of the proteasome inhibitors was confirmed by performing analogous studies with Proteasome-GLO (Figure S7a). These results provided strong evidence that enhanced proteasomal degradation was not responsible for the loss of fLuc activity in STS-treated cells.

**Table 1:** Reagents, manufacturers and working concentrations used in this study.

	Reagent	Manufacturer	Working Concentration
StressInducers	Staurosporine (STS)	Sigma	0–50 µM
	Hydrogen Peroxide (H_2_O_2_)	Fisher	0–10 mM
	Hypoxanthine (HX)	Sigma	50 µM
	Xanthine Oxidase (XO)	Sigma	25 mU/mL
Proteasome Inhibitors	MG-132	Fisher	20 µM
	Epoxomicin	Enzo Life Sciences	10 µM
	Lactacystin	Sigma	10 µM
ProteaseInhibitors	Calpain Inhibitor III	Calbiochem	100 µM
	Pepstatin A	Enzo Life Sciences	100 µM
	Ammonium Chloride	Alfa Aesar	1 mM
	z-vad-fmk	Sigma	1 µM
O_2_^•-^scavengers	Tiron	Sigma	10 mM
	TEMPOL	Sigma	10 mM
	MnTMPyp	Enzo Life Sciences	100 µM
^•^OH scavengers	Mannitol	Sigma	100 mM
	DFO	Calbiochem	50 µM
	TEPA	Sigma	50 µM
H_2_O_2_-related Inhibitors	Catalase	Sigma	40 U/mL
	Allopurinol	MP Biomedicals	100 µM
	Acetylsylic Acid (Aspirin)	Fisher	1 mM

Next, inhibitors for various proteases associated with apoptosis and lysosomal degradation were evaluated, namely calpain Inhibitor III (inhibits calpains), Pepstatin A (inhibits aspartyl proteases), ammonium chloride (inhibits phagosome-lysosome fusion), and z-vad-fmk (pan caspase inhibitor). As shown in Figure 3b, none of these inhibitors significantly affected the Rluc8:fLuc ratio of STS-treated cells. The efficacy of the various protease inhibitors was confirmed by appropriate commercial assays (Figure S7b). While the increase in the Rluc8:fLuc ratio observed upon use of the Calpain Inhibitor III on untreated (–STS) cells requires further exploration, one possible explanation may involve the decrease of intracellular antioxidant glutathione (GSH) levels and the associated increase in oxidative stress that has been shown to occur with the addition of this inhibitor[18].

Once the proteasome and various proteases were shown to have insignificant effects on the mechanism responsible for the increase in the Rluc8:fLuc ratio in STS-treated cells, focus was shifted to three oxygen byproducts associated with oxidative stress, i.e. superoxide (O_2_^•-^), hydroxyl radical (^•^OH) and hydrogen peroxide (H_2_O_2_). Three O_2_^•-^ scavengers were tested - Tiron, TEMPOL (both cell-permeable O_2_^•-^ scavengers) and MnTMPyP (a cell-permeable superoxide dismutase (SOD) mimetic) - for their ability to reduce the Rluc8:fLuc ratio by rescuing fLuc activity. As shown in Figure 3c, none of the superoxide scavengers significantly reduced the Rluc8:fLuc ratio. The efficacy of the O_2_^•-^ scavengers was confirmed by performing a dihydroethidium assay (Figure S7c).

Next, three ^•^OH scavengers/inhibitors, namely mannitol (specific ^•^OH scavenger), deferoxamine (DFO, iron chelator) and tetraethylenepentamine (TEPA, copper chelator) were examined. The two metal chelators, alone and in combination, were used to effectively reduce the amounts of metals available for the Fenton reaction (Fe^2+^ + H_2_O_2_ → Fe^3+^ + ^•^OH + OH^−^). Interestingly, none of the ^•^OH scavengers/inhibitors provided protection for fLuc activity. Accordingly, these agents did not reduce the Rluc8:fLuc ratio in STS-treated cells (Figure 3d). It may be argued that the slight increase in Rluc8:fLuc ratio that was observed following the addition of TEPA to the HeLa-fR cells could be a result of excess H_2_O_2_ buildup from the prevention of the Fenton reaction, however further studies are warranted especially since the combination of DFO + TEPA did not yield an additive effect. The effective reduction of intracellular ^•^OH following the addition of each scavenger/inhibitor was confirmed by a decrease in hydroxyphenyl fluorescein fluorescence (Figure S7d).

The final group of inhibitors/scavengers that were examined was associated with reducing intracellular H_2_O_2_ levels, i.e. catalase (scavenger), allopurinol (xanthine oxidase (XO) inhibitor), and acetylsalicylic acid (aspirin, cyclooxygenase (COX) inhibitor). Catalase converts H_2_O_2_ to water, allopurinol inhibits XO, an enzyme that catalyzes the oxidation of hypoxanthine and xanthine, creating H_2_O_2_ as a byproduct, and aspirin inhibits COX enzymes, which possess peroxidase activity. The addition of all three of these reagents resulted in significant reduction (p<0.01) in the Rluc8:fLuc ratio, as seen in Figure 3e. It should be noted that catalase cannot cross cell membranes, but our results are consistent with previous studies reporting that extracellularly added catalase is effective at removing intracellular H_2_O_2_, likely by creating an extracellular sink [19], [20]. A reduction in intracellular H_2_O_2_ following the addition of each inhibitor/scavenger was confirmed by a decrease in CM-H_2_DCFDA fluorescence (Figure S7e).

The potential involvement of H_2_O_2_ in the loss in fLuc activity in STS-treated cells is consistent with earlier findings. For example, the higher molecular weight fLuc bands that were observed in Figure 2 could be the result of peroxidase-catalyzed H_2_O_2_–dependent cross-linking. Further, the increase in the RLuc8:fLuc ratio that was observed upon pre-treating stressed cells with proteasome inhibitors could also be explained by the H_2_O_2_-medited reduction in fLuc activity since proteasome inhibition has previously been associated with an increase intracellular ROS levels [21], [22], [23], [24]. Perhaps, it was somewhat surprising that the superoxide scavengers TIron, TEMPOL and MnTMPyP resulted in little to no increase in the RLuc8:fLuc ratio considering that superoxide is a precurser of H_2_O_2_; however, measurements of H_2_O_2_ via CM-H_2_DCFDA fluorescence also indicated that Tiron, TEMPOL, and MnTMPyP had no statistically significant effect on H_2_O_2_ levels in STS-treated cells (Figure S8).

### Catalase, allopurinol and aspirin rescue fLuc in a dose-dependent manner and rescue fLuc protein levels in STS treated cells

**Figure 4:**
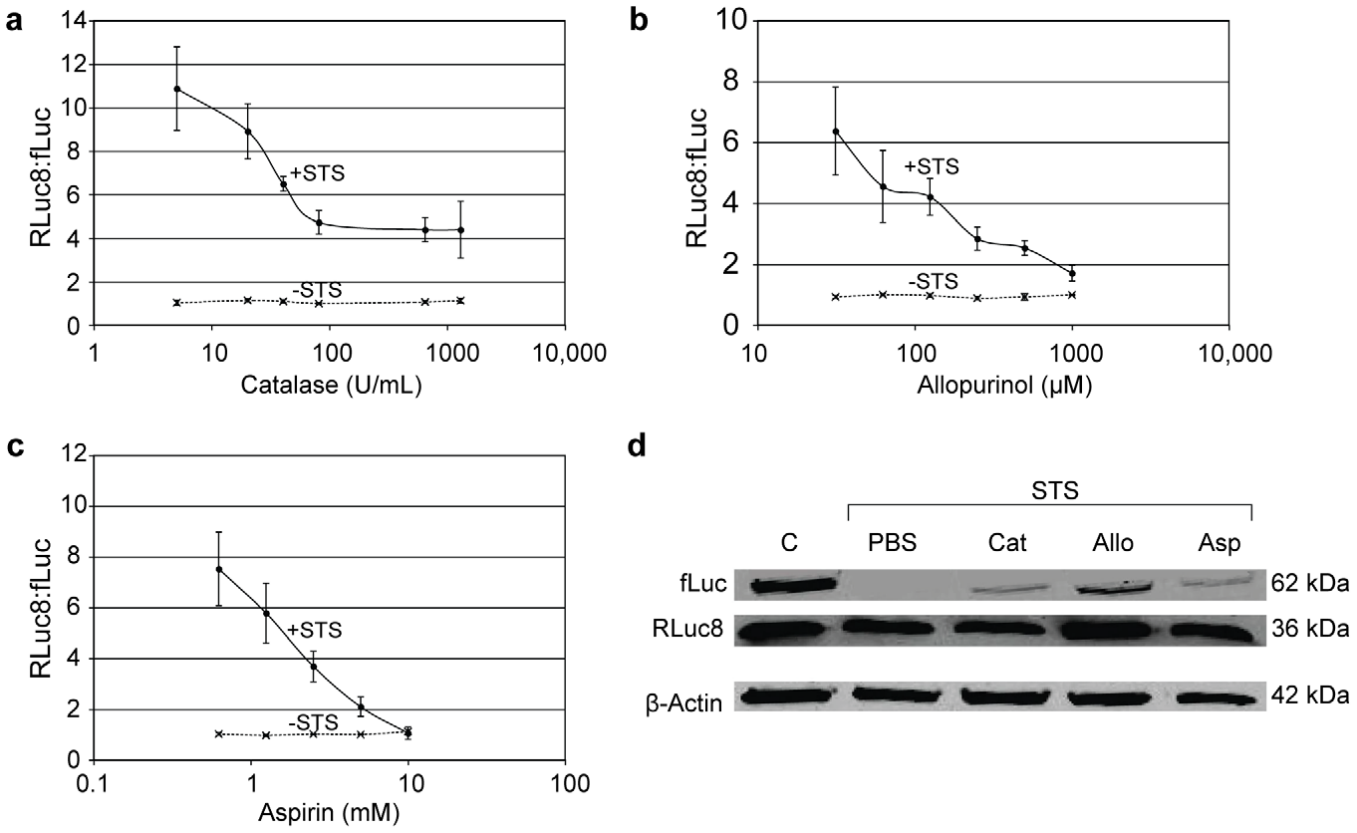
Dose response of HeLa-fR cells to H_2_O_2_-related scavengers/inhibitors. HeLa-fR cells were pretreated with a dosage range of (a) catalase, (b) allopurinol, or (c) aspirin for 1 hour prior to incubating with 10 µM STS (solid lines, +STS) or PBS (dotted lines, -STS) for 24 hours. The RLuc8:fLuc ratio was calculated and reported. (d) HeLa-fR cells were pretreated for 1 hour with PBS, 50 U/mL catalase, 100 µM allopurinol or 1 mM aspirin (columns 2–5) prior to the administration 10 µM STS for 24 hours. A western blot was subsequently performed with anti-fLuc and anti-RLuc antibodies. β-actin is shown as a loading control.

To validate the protective effect of H_2_O_2_ inhibitors/scavengers on fLuc activity, HeLa-fR cells were pretreated with increasing doses of catalase (Figure 4a), allopurinol (Figure 4b) or aspirin (Figure 4c) for 1 hour prior to the addition of 10 µM STS. After a 24 hr incubation period with the various H_2_O_2_ inhibitors/scavengers and STS, the bioluminescent ratio was measured. It was found that each inhibitor/scavenger effectively rescued fLuc activity, thus reducing the Rluc8:fLuc ratio, in a dose-dependent manner. A Western blot was also performed to directly determine the effect of the various H_2_O_2_ inhbitors/scavengers on bioluminescent protein levels. Consistent with the observed recovery in fLuc activity, STS-treated HeLa-fR cells that were pretreated with catalase, allopurinol, or aspirin exhibited higher levels of fLuc protein (Figure 4d, column 3–5) compared to cells treated with STS in the absence of inhibitor (i.e. PBS, column 2).

### Allopurinol can rescue fLuc activity, independent of cell death

**Figure 5:**
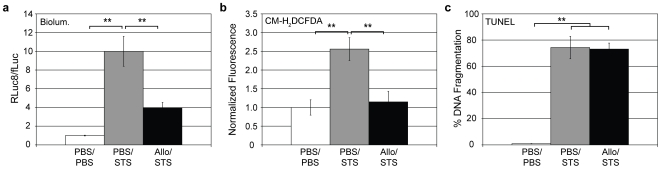
Effect of allopurinol pretreament on STS-treated HeLa cells. HeLa-fR cells were pretreated with either 100 µM allopurinol or PBS for 1 hour prior to the administration of 10 µM STS or PBS. After 24 hours, (a) the RLuc8:fLuc ratio, (b) intracellular H_2_O_2_ levels as measured by CM-H_2_DCFDA, and (c) DNA fragmentation levels, as measured using a TUNEL assay, were recorded for each sample. Statistical significance: *(p<0.05), **(p<0.01).

Although pretreating HeLa-fR cells with allopurinol, prior to the addition of STS, helped decrease the Rluc8:fLuc ratio by stabilizing fLuc activity (Figure 5a) and reducing intracellular H_2_O_2_ levels as determined by CM-H_2_DCFDA (Figure 5b), TUNEL assays revealed that allopurinol did not provide protection against cell death (Figure 5c). These findings demonstrate that the processes associated with fLuc inactivation are separate and distinct from cell death, further supporting the notion that the various proteases that are activated/upregulated in apoptotic cells are not responsible for fLuc inactivation. Moreover, considering that allopurinol specifically inhibits xanthine oxidase, a significant source of hydrogen peroxide (and superoxide and hydroxyl radicals to a lesser extent), these findings provide strong evidence that ROS are primarily responsible for fLuc inactivation in apoptotic cells. It is also interesting to note that since significant reductions in the intracellular level of H_2_O_2_ did not prevent cell death, these results also indicate that H_2_O_2_ is not essential for the progression of programmed cell death in STS-treated HeLa cells, but rather is a downstream byproduct.

### fLuc activity in cells is highly responsive to H_2_O_2_

**Figure 6:**
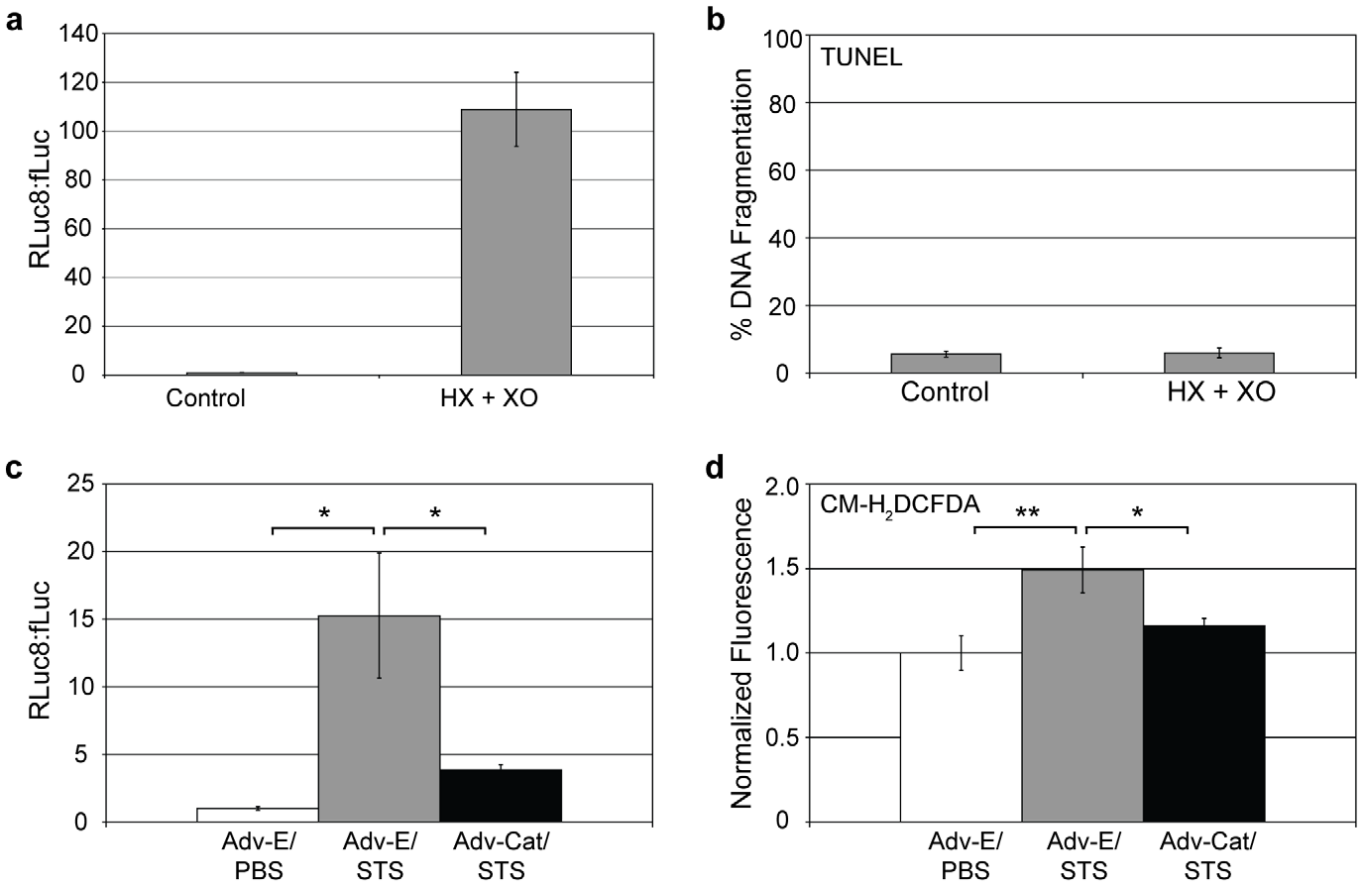
Response of HeLa-fR cells to a hypoxanthine (HX)-xanthine oxidase (XO) reaction and the affect of adenovirus-catalase on STS-treated cells. (a) HeLa-fR cells were subjected to the HX-XO reaction (50 µM HX, 25 mU/mL XO) or PBS (Control) for 24 hours. The RLuc8:fLuc ratio was calculated and reported. (b) A TUNEL assay was used to determine the percent DNA fragmentation, following exposure to HX-XO. (c) HeLa-fR cells were treated with adenovirus-catalase (Adv-Cat) or empty adenovirus (Adv-E) for 24 hours, washed, and incubated in serum-containing media for another 24 hours. Then, 10 µM STS or PBS was added to the cells. After 24 hours the RLuc8:fLuc ratio was calculated and reported. (d) HeLa-fR cells were subjected to the same conditions as in (c) but assayed for H_2_O_2_ levels using CM-H_2_DCFDA.

Having shown that various inhibitors/scavengers of H_2_O_2_ exhibited a stabilizing affect on fLuc activity in apoptotic cells, additional studies were performed that dealt with H_2_O_2_ more directly. First, we investigated the response of HeLa-fR cells to the hypoxanthine (HX)-XO reaction, which allowed for the continual extracellular production of H_2_O_2_. After 24 hours, it was found that the bioluminescent ratio increased dramatically, reaching levels that were significantly higher than what was observed previously with STS treatment (Figure 6a). Additionally, it was found that the HX-XO reaction did not cause cell death, as indicated by a TUNEL assay (Figure 6b). These results provide additional evidence that the observed loss in fLuc activity is specifically associated with elevated levels of ROS as opposed to other mechanisms that are a consequence of cell death.

Second, to specifically reduce H_2_O_2_ in apoptotic cells, HeLa-fR cells were infected with an adenoviral vector coding for catalase, prior to STS treatment. Overexpression of catalase led to a dramatic improvement in the stability of fLuc activity in STS-treated HeLa-fR cells and a corresponding decrease in the Rluc8:fLuc ratio, compared with control cells (i.e. cells treated with STS and empty adenovirus) (Figure 6c). A reduction in intracellular H_2_O_2_ in cells pretreated with adenovirus-catalase was confirmed by a decrease in CM-H_2_DCFDA fluorescence (Figure 6d).

### fLuc is not degraded by the proteasome secondary to inactivation

**Figure 7:**
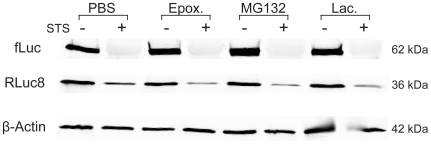
Assessment of fLuc and RLuc8 protein levels in HeLa-fR cells pre-treated with proteasome inhibitors prior to the administration of STS. HeLa-fR cells were pretreated for 1 hour with PBS, 20 µM MG-132, 10 µM Epoxomicin or 10 µM Lactacystin prior to the administration of PBS (-) or 10 µM STS (+) for 24 hours. A western blot was subsequently performed with anti-fLuc and anti-RLuc antibodies. β-actin is shown as a loading control.

It is widely acknowledged that oxidatively-modified proteins can be targeted to the proteasome for degradation [25]. To investigate whether the proteasome was ultimately responsible for the degradation of fLuc following ROS-mediated inactivation in STS-treated cells, a western blot was performed on cells that were pretreated with the proteasome inhibitors MG-132, epoxomicin or lactacystin prior to the administration of STS. As shown in Figure 7, none of the proteasome inhibitors examined were capable of preventing fLuc degradation. These findings provide strong evidence that fLuc was not degraded by the proteasome secondary to inactivation. This leaves open the possibility that oxidatively-modified proteins are specifically degraded via other proteases; however, this seems unlikely considering the degradation of oxidatively-modified proteins is generally a well accepted physiological function of the proteasomal system, with proteolysis by the 20S proteasome being the major pathway.[17], [25], [26] Taking this into consideration, we suspect that ROS may be directly responsible for the degradation of fLuc. It has been well documented that peptide bond cleavage can occur as a result of ROS attack.[27], [28] The formation of protein aggregates and/or cross-linking of fLuc may also contribute to the apparent loss in the wild-type fLuc band on western blots of STS-treated cells,[25] but it is hypothesized that protein cleavage is the predominant mechanism considering that cross-linking was only observed on western blots after long exposures to STS (Figure 2).

## Discussion

In this study, we found that fLuc activity was rapidly lost in apoptotic cells, while RLuc8 exhibited significantly prolonged functionality under the same conditions. Inhibition assays that targeted intracellular protein degradation/modification pathways that are often involved in apoptosis suggested that ROS, primarily H_2_O_2_, were responsible for the rapid loss in fLuc activity. Consistent with these findings, allopurinol, an inhibitor of xanthine oxidase-a primary source of intracellular ROS, aspirin, a COX inhibitor, and catalase, an H_2_O_2_ scavenger, all exhibited a dose-dependent protective effect on fLuc activity in apoptotic cells. Notably, none of these H_2_O_2_ scavengers/inhibitors interfered with cell death, providing further evidence that the proteases and degradation pathways triggered with STS treatment and necessary for cell death were not responsible for the loss in fLuc activity, since these mechanisms will have remained activated after antioxidant treatment.

To directly demonstrate that oxidative stress could lead to the preferential loss of fLuc activity, compared with RLuc8, HX-XO was used to generate H_2_O_2_ in the media of HeLa-fR cells. As expected, the bioluminescent ratio, RLuc8:fLuc, increased dramatically. Importantly, these studies were conducted at H_2_O_2_ levels that were below the threshold needed to cause cell death. Therefore, a state of oxidative stress was created that was independent of any processes associated with cell death.

Currently, it remains unclear whether amino acid modifications or peptide cleavage, as a result of ROS attack, is ultimately responsible for the loss in fLuc activity. Certainly, both mechanisms could play a role. Protease-mediated degradation of oxidatively modified proteins can also not be ruled out completely; however, considering that selective degradation of oxidatively-modified proteins is the physiological function of the proteasomal system[17], [25], [26] and inhibition of this system did not prevent the loss of the fLuc band on western blots, it seems more likely that any degradation observed was the direct result of ROS attack.

Common targets for H_2_O_2_-mediated modifications include but are not limited to methionine (M), cysteine (C), histidine (H), tyrosine (Y) and phenylalanine (F), (see [17], for review), often creating hydroxyl- or carbonyl-derivatives. Glutamyl (E), aspartyl (D), and prolyl (P) side chains are common sites for peptide bond cleavage as a result of oxidative stress. Since the proposed active site (i.e. luciferin binding site) for fLuc is 244H-H-G-F247 [29], there are a number of potential amino acid substrates for oxidative modification. However, it should be noted that the proposed active site for rLuc, D120, E144, and H285, also contains multiple targets for ROS [14]. Therefore, it not clear whether the differential sensitivity of fLuc and RLuc8 to H_2_O_2_ can be attributed to modifications within these proposed active sites alone. Further, there is still some uncertainty regarding the exact amino acids of fLuc that are involved in substrate binding and enzyme catalysis [30], [31]. Other amino acids that have been identified as being important for bioluminescence include S198, K206, Y340, T343, E344, E389 and Y401[32]. It is possible that the H_2_O_2_-mediated modification of various amino acids and/or combinations of amino acids could all lead to the inactivation of fLuc. Certainly, peptide cleavage at any location within the amino acid sequence of fLuc could lead to inactivation of fLuc, as well. In fact, it has been shown that even just the removal of a few amino acids from the N- or C-terminus from fLuc can cause a dramatic loss in fLuc activity.[33], [34] Future studies (e.g. mass spectroscopy, mutagenesis, and functionality assays) will be aimed at analyzing various location(s) and types of amino acid modifications and cleavage products to determine which could lead to a diminution of fLuc activity.

Despite the uncertainty regarding the specific amino acid modifications or cleavage sites responsible for the inactivation of fLuc, we have shown here that the bioluminescent ratio RLuc8:fLuc can still be used to relay important information regarding intracellular levels of ROS, in particular hydrogen peroxide [35], [36]. For example, we showed that the increase in the level of intracellular H_2_O_2_ in various cases of cell death was consistently reflected by an increase in the RLuc8:fLuc ratio. Of course, these studies do not rule out the possibility that other factors could also have a differential effect on fLuc and RLuc; however, the contribution of ROS to the loss of fLuc activity can be established by performing control studies with H_2_O_2_-specific scavengers/inhibitors.

Arguably, measurements of RLuc8:fLuc ratio could thus be used to assist with the identification and evaluation of any cancer therapeutics that induce elevated levels of ROS. This is likely to include compounds that induce cell death through caspase-dependent and caspase-independent pathways [10], [22], [36], [37], [38], [39], [40], [41]. Applications related to caspase-independent cell death are of particular interest considering that anticancer drug resistance and tumorigenesis have been linked to the ability of certain cancer types to evade caspase activation [42], [43]. Currently, there is a dearth of tools available for the study of caspase-independent cell death. Therefore, having a bioluminescent read-out that can potentially be translated from benchtop assays to *in vivo* disease models is particularly beneficial. With an increasing number of pathologies being linked to oxidative stress, including atherosclerosis, cancer, cystic fibrosis, type-2 diabetes, and Alzheimer's disease, the ability to detect oxidative stress is also expected to allow for advances in anti- and pro-oxidant research across a wide range of disciplines.

## Materials and Methods

### Plasmid Vector Construction

The internal ribosome entry site (IRES) from the pIRES2-DsRed Express Vector (Clontech, Mountain View, CA, USA) was cloned into the pIRES vector (Clontech) using restriction enzymes NheI and KpnI (New England Biolabs, Ipswich, MA, USA), creating the pIRES12 vector. The phRL-CMV vector (Promega, Madison, WI, USA) encoding Renilla Luciferase was modified to contain eight amino acid mutations within the rLuc sequence (A55T, C124A, S130A, K136R, A143M, M185V, M253L, and S287L) using the QuikChange Multi Site Directed Mutagenesis Kit (Stratagene, La Jolla, CA, USA) according to the manufacturer's instructions, ultimately creating RLuc8. The DNA sequence encoding Firefly Luciferase (fLuc) from the pGL3-Basic vector (Promega) was PCR amplified and inserted into pCDNA3.1+ (Invitrogen, Carlsbad, CA, USA) between the BamHI and EcoRI restriction sites. The fLuc sequence was subsequently cloned into pIRES12 using the NheI and XbaI restriction sites of fLuc-pCDNA3.1+ and the XbaI site of pIRES12. RLuc8 was cloned into the pIRES12-fLuc vector at the NheI and BglII restriction sites. The resulting Rluc8-IRES-fLuc sequence was cloned into the pLENTI6/V-5 TOPO vector (Invitrogen) per the manufacturer's instructions to create the fLuc-RLuc8 (fR) vector.

### Cell Culture

Human cervical carcinoma (HeLa) and human breast adenocarcinoma (MCF7) cells (ATCC, Manassas, VA) were grown in Eagle's Minimum essential medium (Mediatech, Manassas, VA) supplemented with 10% fetal bovine serum (FBS, HyClone, Logan, UT), 1.5 g/L sodium bicarbonate, 100 U/mL penicillin and 100 µg/mL streptomycin (Invitrogen). Human embryonic kidney (293T/17) cells (ATCC) were grown in Dulbecco's Modified Eagle's Medium (DMEM, Mediatech) supplemented with 10% FBS (Hyclone), 1.5 g/L sodium bicarbonate, 100 U/mL penicillin and 100 µg/mL streptomycin (Invitrogen). The genetically modified human embryonic kidney cells (293FT, Invitrogen) for generating lentiviral particles were cultured in Dulbecco's Modified Eagle's Medium (DMEM, Mediatech) supplemented with 10% fetal bovine serum (FBS, HyClone, Logan, UT), 1.5 g/L sodium bicarbonate, 1 mM sodium pyruvate, 0.1 mM MEM non-essential amino acids (NEAA), 6 mM L-glutamine, 100 U/mL penicillin and 100 µg/mL streptomycin (Invitrogen). Cells that were genetically engineered to stably express the fR vector (described below) also had Blasticidin (Invitrogen) added at a final concentration of 4 µg/mL. All cells were cultivated in a 37°C humidified incubator with 5% CO_2_.

### Lentiviral Particle Production and Stable Cell Line Creation

Lentiviral particles containing the fR vector were produced using the Virapower Lentiviral Directional TOPO Expression Kit (Invitrogen) according to the manufacturer's insctructions. Briefly, 293FT cells were transfected with viral packaging plasmids and the fR lentiviral vector using Lipofectamine 2000 (Invitrogen). Viral supernatant was harvested 48 hours after transfection, concentrated using Peg-it Virus Concentration solution (System Biosciences, Mountain View, CA, USA) and the titer was assessed. Concentrated viral particles were added to HeLa cells, which were subsequently selected for stable genomic integration using Blasticidin (Invitrogen), resulting in HeLa-fR cells.

### Inhibition Assays

To induce cellular stress, HeLa cells were treated with Staurosporine (STS), hydrogen peroxide, or hypoxanthine and xanthine oxidase for 0 to 24 hours at concentrations indicated in Table 1. Stressed and unstressed cells were also incubated with inhibitors that target various intracellular protein degradation/modification pathways. The specific compounds/proteins that were utilized as inhibitors and the respective final working concentrations are also listed in Table 1. Inhibitors were added to cells 1-hr prior to adding inducers of cellular stress and left in the media for the duration of the treatment. Analogous controls were conducted in the absence of stress inducers.

### Apoptosis Induction

To induce apoptosis, cells were treated with staurosporine (STS) (Sigma, St. Louis, MO) in complete medium at indicated concentrations and time points. In studies where allopurinol (Allo, MP Biomedicals) is used as an inhibitor, it was added at a final concentration of 100 µM to cells in complete medium for 1 hour prior to the addition of the death-inducing drugs and remained in the medium throughout the study.

### Cellular Bioluminescence Assays

Unless otherwise noted, bioluminescence assays were performed 24 hours after plating 10,000 HeLa-fR cells/well in a white-walled 96 well tissue culture plate (BD Biosciences, Franklin Lakes, NJ, USA). The Dual-Glo Luciferase Assay System (Promega) was utilized according to the manufacturer's instructions to obtain both fLuc and Rluc8 bioluminescence measurements from an Infinite 200 plate reader (Tecan, Mannedorf, Switzerland). In the case of using MnTMPyP as an inhibitor, the cell media was removed, cells were gently washed and the media was replaced before using the Dual-Glo Luciferase Assay System as it was found that the compound interfered with the bioluminescence measurements.

### Adenovirus Infection

5000 Hela-fR cells/well were plated in a white-walled 96-well tissue culture plate (BD Biosciences). The following day, catalase adenovirus (Adv-Cat, Vector Biolabs, Philadelphia, PA) was added to the cells at an MOI of 200. Cells were incubated at 37°C for one hour, with gentle rocking occurring every 15 minutes. Virus-containing media was then replaced with normal growth media. Cell death and bioluminescence assays were performed beginning on the following day, using methods previously outlined.

### Cell Death Assays

#### Caspase Activity

PCD was induced (as described above) 24 hours after plating 10,000 cells/well in white-walled 96 well tissue culture plates (BD Biosciences). After indicated treatment times, the Caspase 3/7-Glo Assay (Promega) was performed according to the manufacturer's protocol and bioluminescence measurements were obtained from and Infinite 200 plate reader (Tecan). All values were normalized to controls (i.e. pre-treatment values).

### DNA Fragmentation

PCD was induced (as described above) 24 hours after plating 300,000 cells/well in 6 well tissue culture plates (BD Biosciences). After indicated treatment times, TUNEL assays for DNA fragmentation were performed using the TUNEL/ApoBRDU assay kit (Invitrogen) according to the manufacturer's protocol. Percent DNA fragmentation was determined using flow cytometry on a Guava EasyCyte flow cytometer (Guava Technologies, Hayward, CA). Analysis of flow cytometry data was performed using FlowJo software (Treestar, Ashland, OR).

### Quantitative Real Time PCR

Cytoplasmic RNA from HeLa-fR cells cultured under indicated conditions was isolated using the High Pure RNA Isolation Kit (Roche, Mannheim, Germany) and subsequently reverse-transcribed to single-stranded cDNA using a High-Capacity cDNA Reverse Transcription Kit (Applied Biosystems, Foster City, CA, USA) according to each manufacturer's protocol. Quantitative RT-PCR was performed on an ABI PRISM 7300 Sequence detection system using FAM-labeled Taqman primer sets for Rluc8, fLuc and β-actin (as a control) and the Taqman universal PCR Master Mix (Applied Biosytems) according to the manufacturer's protocol.

### Western Blotting

Following indicated culture conditions, HeLa-fR cells were washed 3 times with 1x PBS (pH 7.4). Proteins were extracted using RIPA extraction buffer (50 mM Tris HCL, pH 7.4, 1% Triton X-100, 0.25% Na-deoxycholate, 150 mM NaCl, 1 mM EDTA and a Complete Mini Protease Inhibitor Cocktail Tablet (Roche)) at 4°C for 30 minutes with constant agitation. Total protein concentrations were measured using a BCA assay (Pierce, Rockford, IL, USA). 30 µg of total protein from each sample were heated to 95°C in Laemmli Sample buffer containing 2% (v/v) 2-mercaptoethanol (Bio-Rad, Hercules, CA, USA). After a 5 minute cooling period, the samples were quickly centrifuged and the supernatants were immediately run on a 4–15% Tris-HCl gel (Bio-Rad). Proteins separated by electrophoresis were transferred to nitrocellulose membranes in 1X Transfer Buffer (Bio-Rad) at 15 V for 30 minutes. Membranes were blocked in Blocking Buffer for Fluorescent Western Blotting (Rockland Immunochemicals, Gilbertsville, PA, USA) for 60 minutes. The membranes were incubated with anti-fLuc (Sigma), anti-rLuc (Millipore, Billerica, MA) or anti-β-actin (Abcam, Cambridge, MA) primary antibodies in blocking buffer overnight. After washing 3 times with TBS-T, the membranes were incubated with either Anti-Mouse IgG Antibody IRDye800 conjugated (fLuc, rLuc) or Anti-Rabbit IgG Antibody IRDye800 conjugated (β-actin) at a 1∶10,000 dilution (Rockland Immunochemicals). The fluorescent signal from the membranes was imaged using the Odyssey Infrared Imaging System (Li-Cor Biosciences, Lincoln, NE, USA).

### Cellular Bioluminescence Imaging

HeLa-fR cells were plated at a density of 10,000 cells/well in a black-walled 96 well tissue culture plate (BD Biosciences). The Dual-Glo Luciferase Assay System (Promega) was utilized according to the manufacturer's instructions in order to obtain bioluminescent images in an Omega 16 vs imaging system (UltraLum, Claremont, CA, USA). It should be noted that the exact position of the 96 well plate was kept constant throughout the imaging session in order for accurate ratiometric images to be calculated.

### Microplate Image Analysis

All image analyses were performed using ImageJ (NIH, Bethesda, MD, USA). Image background was determined by measuring 3 regions of interest (ROIs) surrounding the bioluminescent ROI. After background subtraction, the RLuc8 image was divided by the fLuc image using the ‘Math’ command under the ‘Process’ menu tab.

### Assessing Sensitivity of Bioluminescent Substrates to Intracellular Species

HeLa cells were plated at a density of 10,000 cells/well in a white-walled 96-well plate (BD Biosciences). 24 hours later, cells were treated with PBS (pH 7.4) or 10 µM STS. Following a 24 hour incubation, Dual-Glo (Promega) substrates were applied to the cells along with 650 nM purified fLuc and RLuc8 proteins. Bioluminescence measurements were obtained from an Infinite 200 plate reader (Tecan).

### Switching of fLuc and RLuc8 relative to the IRES sequence

The RLuc8 sequence was PCR amplified and cloned into pCDNA3.1+ between the BamHI and EcoRI restriction sites, creating RLuc8-pCDNA2.1+. RLuc8 was subsequently cloned into pIRES12 using the NheI and XbaI restriction sites of RLuc8-pCDNA3.1+ and the XbaI site of pIRES12, creating pIRES12-RLuc8. The fLuc sequence was PCR amplified and inserted into pCDNA3.1+ between the BamHI and EcoRI restriction sites. The fLuc sequence was subsequently cloned into the pIRES12-RLuc8 vector at the NheI and BglII restriction sites. The resulting fluc-pIRES-RLuc8 sequence was cloned into the pLENTI6/V-5 TOPO vector per the manufacturer's instructions to create the final vector necessary for the reverse RBS.

### Proteasome Inhibition Control Assay

HeLa-fR cells were plated at a density of 10,000 cells/well in a white-walled 96-well plate (BD Biosciences). 24 hours later, cells were treated with PBS (pH 7.4), 20 µM MG-132, 10 µM epoxomicin or 10 µM lactacystin for 1 hour. HeLa-fR cells were then treated with PBS (pH 7.4) or 10 µM Staurosporine (STS) in addition to the inhibitors for 24 hours. Proteasome activity measurements were obtained using the Proteasome-Glo 3-Substrate Cell-Based Assay system (Promega) according to the manufacturer's instructions.

### Protease Inhibition Control Assays: Calpain Inhibitor III

HeLa-fR cells were plated at a density of 10,000 cells/well in a white-walled 96-well plate (BD Biosciences). 24 hours later, cells were treated with either PBS (pH 7.4) or 100 µM Calpain Inhibitor III for 1 hour. PBS (pH 7.4) or 10 µM STS was subsequently added to the cells, and both compounds remained on the cells for 24 hours. Calpain activity measurements were obtained using the Calpain-Glo Protease Assay (Promega) according to the manufacturer's instructions.

### Pepstatin A

HeLa-fR cells were plated at a density of 10,000 cells/well in a black-walled 96-well plate (BD Biosciences). 24 hours later, cells were treated with either PBS (pH 7.4) or 100 µM pepstatin A for 1 hour, with either PBS (pH 7.4) or 10 µM STS added for another 24 hours. Cathepsin activity was assessed using the CV-Cathepsin B Detection Kit (Enzo Life Sciences) according to the manufacturer's instructions with one exception; after the final wash step, the plate was read on an Infinite 200 plate reader (Tecan) for fluorescence (550_ex_/610_em_), as opposed to microscopy.

### Ammonium Chloride

In lieu of a control assay for ammonium chloride effectively inhibiting lysosome-phagosome fusion, we direct the reader to a seminal paper regarding this phenomena in HeLa cells [1].

### z-vad-fmk

HeLa-fR cells were plated at a density of 10,000 cells/well in a white-walled 96-well plate (BD Biosciences). 24 hours later, cells were treated with either PBS (pH 7.4) or 1 µM z-vad-fmk for 1 hour. PBS (pH 7.4) or 10 µM STS was subsequently added to the cells, and both compounds remained on the cells for 24 hours. Caspase activity measurements were obtained using the Caspase-Glo 3/7 Assay (Promega) according to the manufacturer's instructions.

### Superoxide (O_2_^•-^) Scavenger Control Assay

HeLa-fR cells were plated at a density of 120,000 cells per well of a 12 well tissue culture plate (BD Biosciences). 24 hours later, cells were treated with PBS (pH 7.4), 10 mM TEMPOL, 10 mM Tiron or 100 µM MnTMPyP for 1 hour. PBS (pH 7.4) or 10 µM STS was subsequently added to the cells, and both compounds remained on the cells for 24 hours. Intracellular O_2_^•-^ levels were then determined by incubating the cells in 5 µM dihydroethidium (DHE, Invitrogen) for 30 minutes at 37°C and subjecting them to flow cytometry using a Guava EasyCyte (Guava Technologies, Hayward, CA, USA). Analysis of flow cytometry data was accomplished using FlowJo software (TreeStar, Ashland, OR, USA).

### Hydroxyl Radical (^•^OH) Scavenger/Inhibitor Control Assay

HeLa-fR cells were plated at a density of 120,000 cells per well of a 12 well tissue culture plate (BD Biosciences). 24 hours later, the cells were incubated with 10 µM hydroxyphenylfluorescein (HPF, Sigma) for 30 minutes at 37°C. Cells were then treated with PBS (pH 7.4), 100 mM Mannitol, 50 µM deferoxamine (DFO), 50 µM tetraethylenepentamine (TEPA) or 50 µM DFO plus 50 µM TEPA for 1 hour. PBS (pH 7.4) or 10 µM STS was subsequently added to the cells, and all compounds remained on the cells for 24 hours. Intracellular ^•^OH levels were determined by subjecting the cells to flow cytometry using a Guava EasyCyte (Guava Technologies). Analysis of flow cytometry data was accomplished using FlowJo software (TreeStar).

### Hydrogen Peroxide (H_2_O_2_)-related Inhibitor Control Assays: Catalase and Allopurinol

HeLa-fR cells were plated at a density of 120,000 cells per well of a 12 well tissue culture plate (BD Biosciences). 24 hours later, the cells were incubated with 10 µM CM-H_2_DCFDA (Invitrogen) for 30 minutes at 37°C. Cells were then treated with PBS (pH 7.4), 50 U/mL catalase or 100 µM allopurinol for one hour. PBS (pH 7.4) or 10 µM STS was subsequently added to the cells, and all compounds remained on the cells for 24 hours. Intracellular H_2_O_2_ levels were determined by subjecting the cells to flow cytometry using a Guava EasyCyte (Guava Technologies). Analysis of flow cytometry data was accomplished using FlowJo software (Treestar).

### Aspirin

HeLa-fR cells were plated at a density of 1.2×10^6^ cells in 100 mM tissue culture dishes (BD Biosciences). 24 hours later, the cells were treated with PBS (pH 7.4) or 1 mM aspirin for one hour, followed by PBS (pH 7.4) or 10 µM STS for 24 hours. Cyclooxygenase (COX) activity was determined by using the Cox Activity Assay Kit (Cayman Chemical Company, Ann Arbor, MI, USA).

### H_2_O_2_ Detection in Cells treated with Superoxide Scavengers

HeLa-fR cells were plated at a density of 120,000 cells per well of a 12 well tissue culture plate (BD Biosciences). 24 hours later, the cells were incubated with 10 µM CM-H_2_DCFDA (Invitrogen) for 30 minutes at 37°C. Cells were then treated with PBS (pH 7.4), 10 mM TEMPOL, 10 mM Tiron or 100 µM MnTMPyP for 1 hour. PBS (pH 7.4) or 10 µM STS was subsequently added to the cells, and both compounds remained on the cells for 24 hours. Intracellular H_2_O_2_ levels were determined by subjecting the cells to flow cytometry using a Guava EasyCyte (Guava Technologies). Analysis of flow cytometry data was accomplished using FlowJo software (Treestar).

## Supporting Information

Figure S1 **Analysis of RLuc8:fLuc ratio as a function of time and cell number.** (**a**) For a fixed cell seeding density, the RLuc8 and fLuc bioluminescent signal that was elicited by HeLa-fR cells (PBS-treated) was detected over the course of 24 hrs (left axis) and the RLuc8:fLuc ratio was calculated at each time point (right axis). (**b**) HeLa-fR cells were plated at various cell densities and the RLuc8:fLuc ratio was measured.
(TIF)

Figure S2 **Caspase 3/7 activity in STS-treated HeLa-fR cells.** (**a**) HeLa-fR cells were treated with 10 µM STS or PBS (untreated) for up to 24 hours. A Caspase-Glo 3/7 assay (Promega) was performed at various times during the course of treatment. All measurements were normalized to values at 0 hours. (**b**) HeLa-fR cells were treated with 0–50 µM STS of PBS (untreated) for 6 hours followed by a Caspase-Glo 3/7 assay (Promega). All measurements were normalized to values at 0 µM STS.
(TIF)

Figure S3 **Response of MCF7 and 293T/17 cells to increasing doses of STS.** (**a**–**c**) MCF7-fR and (**d**–**f**) 293T/17-fR cells were treated with a dosage range of STS (0–50 µM) for 6 hours. (**a,d**) Bioluminescent measurements of RLuc8 and fLuc were acquired for each STS concentration after 6 hours (left axis). The RLuc8:fLuc ratio was subsequently calculated for each STS concentration (right axis). (**b,e**) A TUNEL assay for DNA fragmentation was performed to provide a measure of cell death. (**c,f**) Caspase 3/7 activity was determined using a Caspase 3/7 Glo assay. In all studies (except TUNEL assays, which provide an absolute measure of cell death), measurements were normalized to values at 0 µM STS.
(TIF)

Figure S4 **Measurements of the bioluminescent ratio after the coding sequences of fLuc and RLuc8 were interchanged, relative to the IRES sequence.** HeLa-fR cells were treated with PBS or 10 µM STS for 24 hours and bioluminescent measurements of RLuc8 and fLuc were acquired. The ratio RLuc8:fLuc was subsequently calculated.
(TIF)

Figure S5 **Assessment of D-luciferin and coelenterazine stability in the presence of intracellular species and enzymes.** Wild-type HeLa cells were treated either with STS or PBS for 24 hours. Subsequently, both D-luciferin and coelenterazine (in lysis buffer – Dual Glo) were added to the cells and this was followed by the addition of purified fLuc and RLuc8 proteins. Bioluminescent measurements of fLuc and RLuc8 were acquired and normalized to the mean of PBS-treated controls, in the absence of STS.
(TIF)

Figure S6 **Schematic representing potential pathways for intracellular protein modification and/or degradation.**
(TIF)

Figure S7 **Control assays to validate inhibitor effectiveness.** HeLa-fR cells were pretreated with either PBS (gray bars) or indicated inhibitors (white bars) for 1 hour before the addition of 10 µM STS. The PBS/Inhibitors and STS were then incubated with the cells for an additional 24 hours. (**a**) Proteasome inhibitors were assayed using Proteasome-Glo. (**b**) Protease inhibitors were assayed using Calpain-Glo (Calpain Inhibitor III), CV-Cathepsin B Detection Kit (Pepstatin A) or Caspase-Glo 3/7 (z-vad-fmk). (**c**) O_2_^•-^ scavengers were assayed using DHE. (**d**) ^•^OH scavengers were assayed using HPF. (**e**) H_2_O_2_-related scavengers were assayed using CM-H_2_DCFDA (catalase and allopurinol) or the Cox Activity Assay Kit (aspirin). All measurements were normalized to the mean measurements in the absence of the respective inhibitor. Statistical significance between -inhibitor and +inhibitor is indicated with a * (p<0.05) or ** (p<0.01).
(TIF)

Figure S8 **Measurements of H_2_O_2_ in HeLa-fR cells pretreated with superoxide scavengers prior to the addition of STS.** HeLa-fR cells were pretreated with PBS (control), 10 mM Tiron, 10 mM TEMPOL, or 100 µM MnTMPyp for 1 hour, followed by PBS (white bars) or 10 µM STS (gray bars) for 24 hours. HeLa cells were then assayed for H_2_O_2_ levels using CM-H2DCFDA. All measurements were normalized to the mean of PBS-treated controls, in the absence of STS.
(TIF)
