# Supplementary figures and images for: Firefly Luciferase and Rluc8 Exhibit Differential Sensitivity to Oxidative Stress in Apoptotic Cells
Source: PLoS One. 2011 May 13;6(5):e20073. doi: 10.1371/journal.pone.0020073 (PMC3094452; doi:10.1371/journal.pone.0020073)

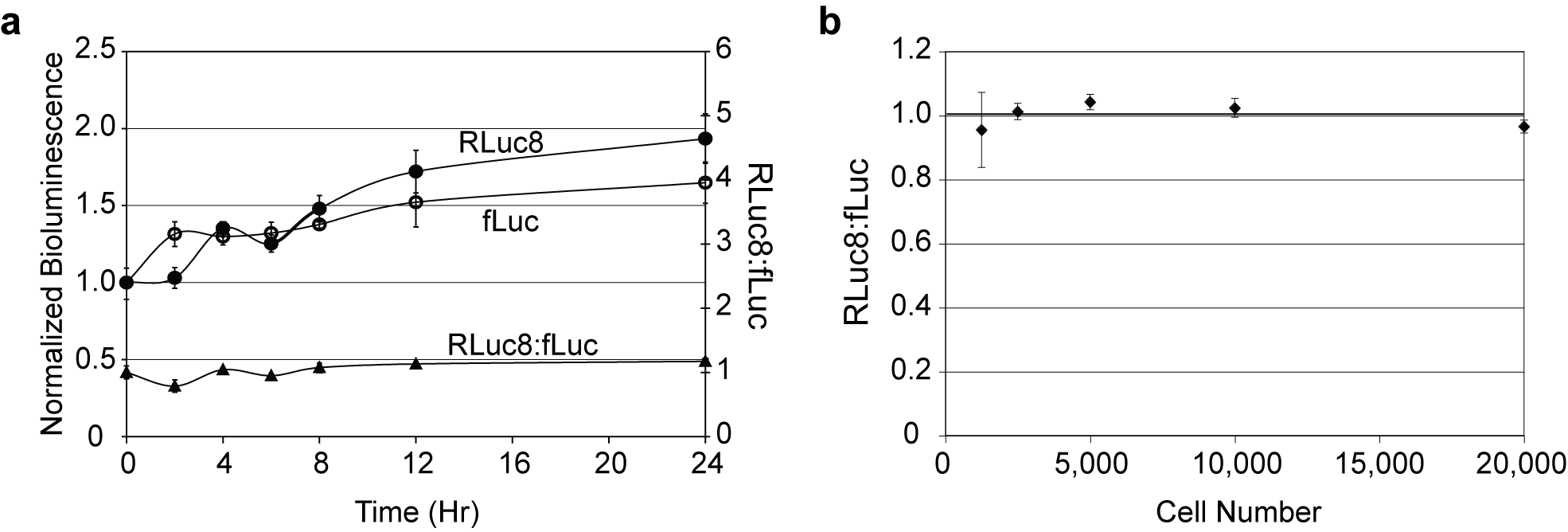

Supplement: Figure S1 — Analysis of RLuc8:fLuc ratio as a function of time and cell number. (a) For a fixed cell seeding density, the RLuc8 and fLuc bioluminescent signal that was elicited by HeLa-fR cells (PBS-treated) was detected over the course of 24 hrs (left axis) and the RLuc8:fLuc ratio was calculated at each time point (right axis). (b) HeLa-fR cells were plated at various cell densities and the RLuc8:fLuc ratio was measured. (TIF) [file pone.0020073.s001.tif]

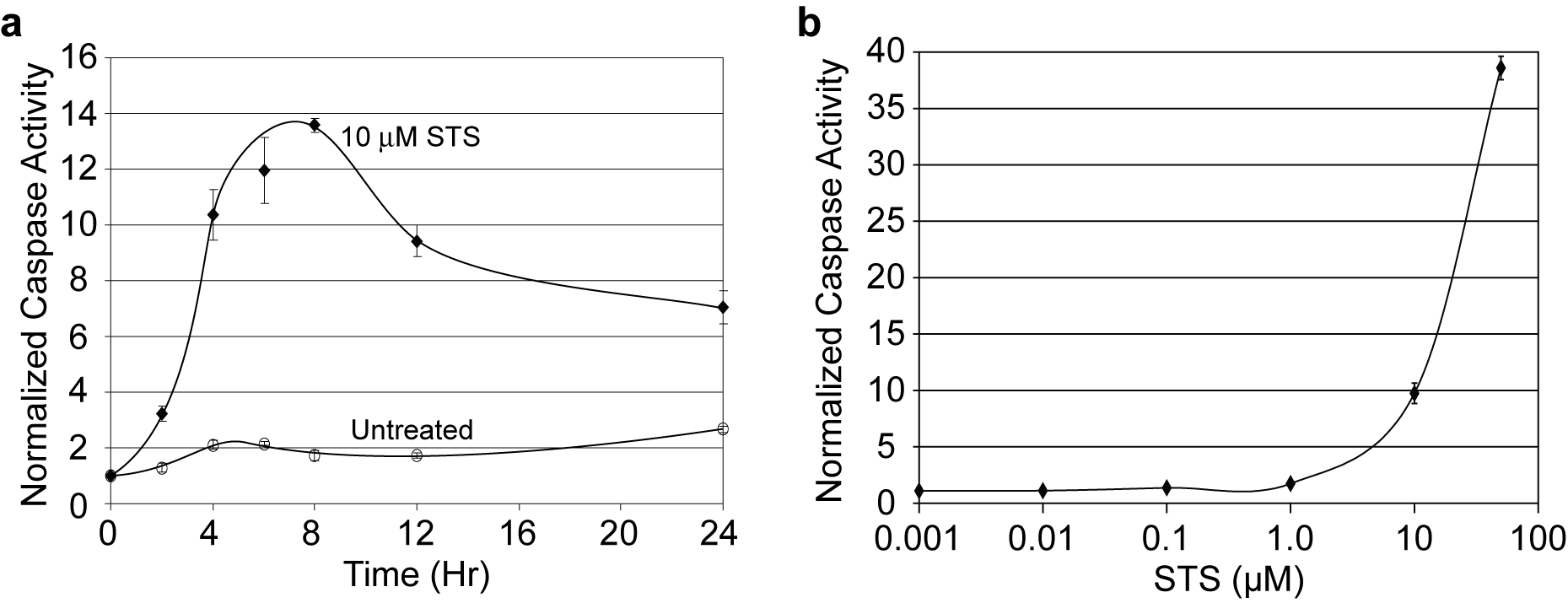

Supplement: Figure S2 — Caspase 3/7 activity in STS-treated HeLa-fR cells. (a) HeLa-fR cells were treated with 10 µM STS or PBS (untreated) for up to 24 hours. A Caspase-Glo 3/7 assay (Promega) was performed at various times during the course of treatment. All measurements were normalized to values at 0 hours. (b) HeLa-fR cells were treated with 0–50 µM STS of PBS (untreated) for 6 hours followed by a Caspase-Glo 3/7 assay (Promega). All measurements were normalized to values at 0 µM STS. (TIF) [file pone.0020073.s002.tif]

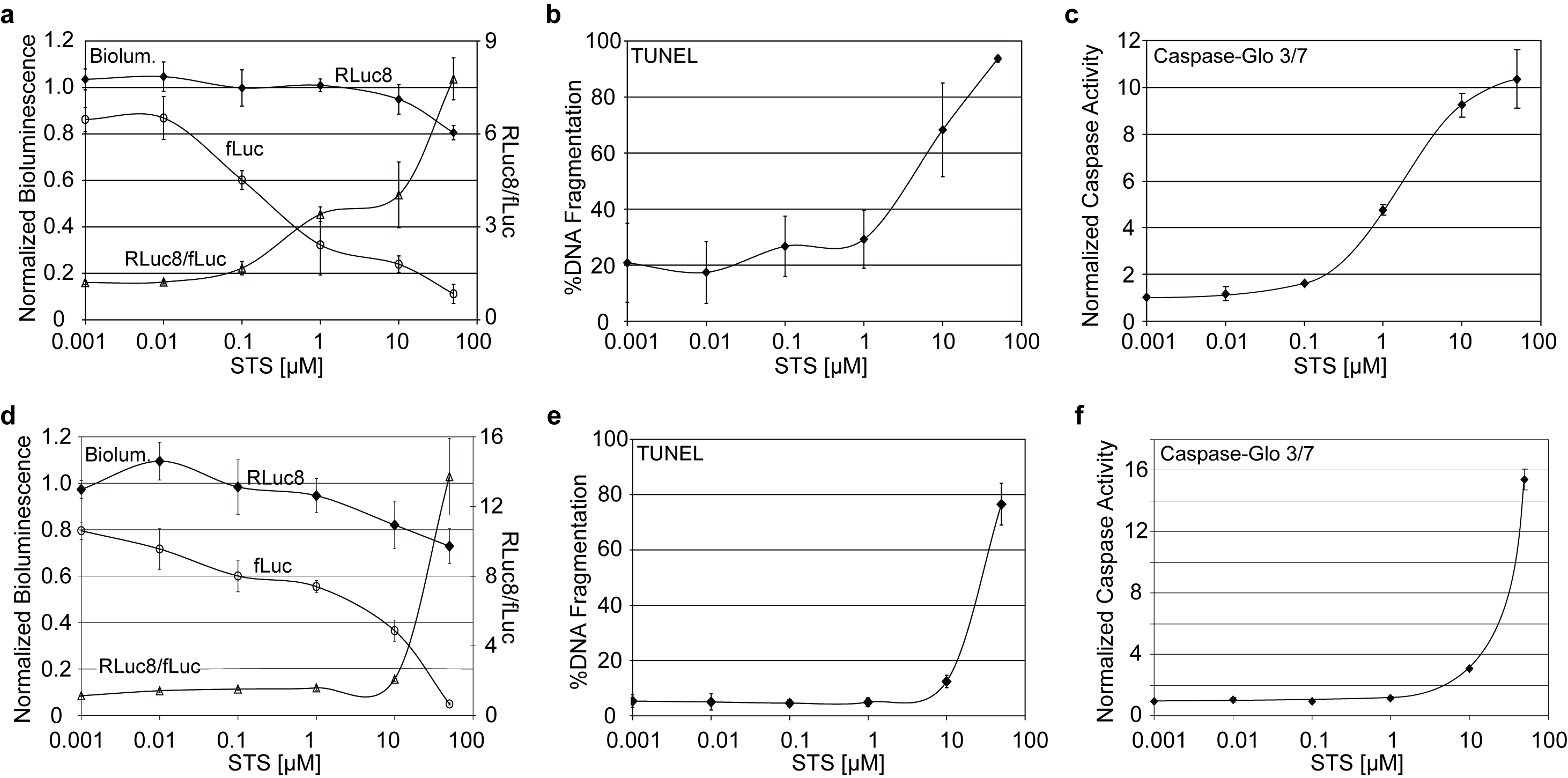

Supplement: Figure S3 — Response of MCF7 and 293T/17 cells to increasing doses of STS. (a–c) MCF7-fR and (d–f) 293T/17-fR cells were treated with a dosage range of STS (0–50 µM) for 6 hours. (a,d) Bioluminescent measurements of RLuc8 and fLuc were acquired for each STS concentration after 6 hours (left axis). The RLuc8:fLuc ratio was subsequently calculated for each STS concentration (right axis). (b,e) A TUNEL assay for DNA fragmentation was performed to provide a measure of cell death. (c,f) Caspase 3/7 activity was determined using a Caspase 3/7 Glo assay. In all studies (except TUNEL assays, which provide an absolute measure of cell death), measurements were normalized to values at 0 µM STS. (TIF) [file pone.0020073.s003.tif]

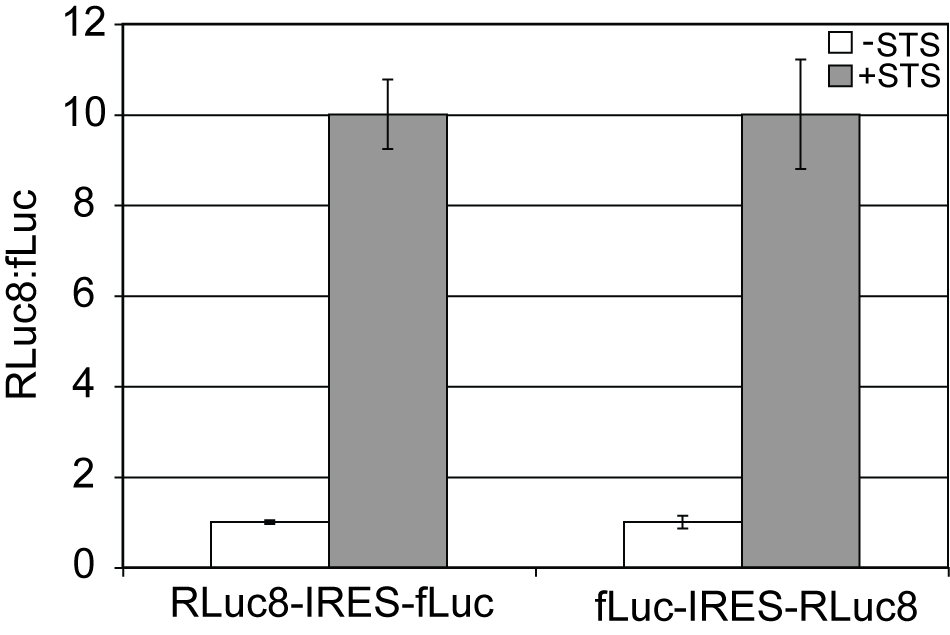

Supplement: Figure S4 — Measurements of the bioluminescent ratio after the coding sequences of fLuc and RLuc8 were interchanged, relative to the IRES sequence. HeLa-fR cells were treated with PBS or 10 µM STS for 24 hours and bioluminescent measurements of RLuc8 and fLuc were acquired. The ratio RLuc8:fLuc was subsequently calculated. (TIF) [file pone.0020073.s004.tif]

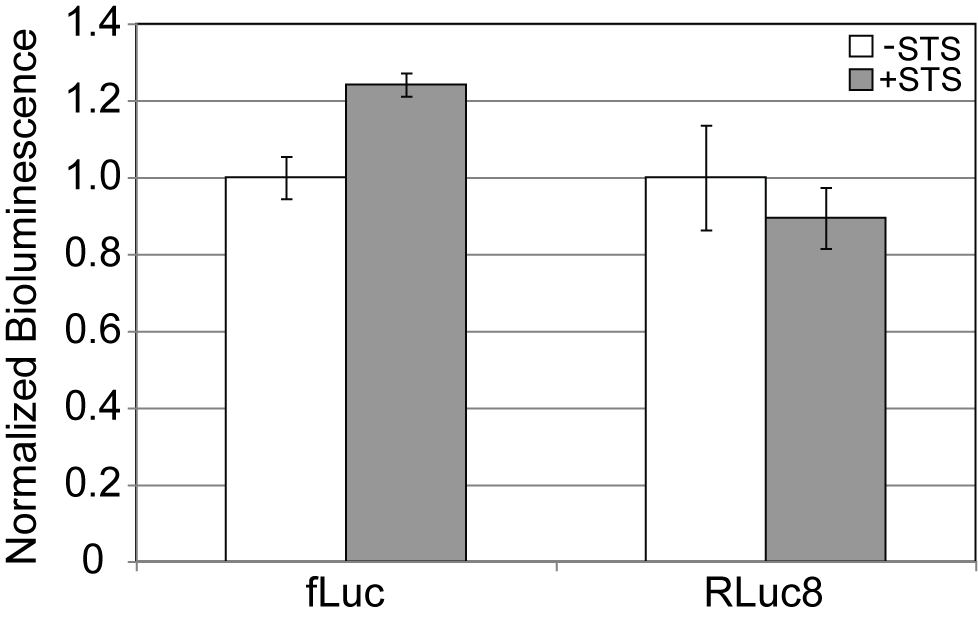

Supplement: Figure S5 — Assessment of D-luciferin and coelenterazine stability in the presence of intracellular species and enzymes. Wild-type HeLa cells were treated either with STS or PBS for 24 hours. Subsequently, both D-luciferin and coelenterazine (in lysis buffer – Dual Glo) were added to the cells and this was followed by the addition of purified fLuc and RLuc8 proteins. Bioluminescent measurements of fLuc and RLuc8 were acquired and normalized to the mean of PBS-treated controls, in the absence of STS. (TIF) [file pone.0020073.s005.tif]

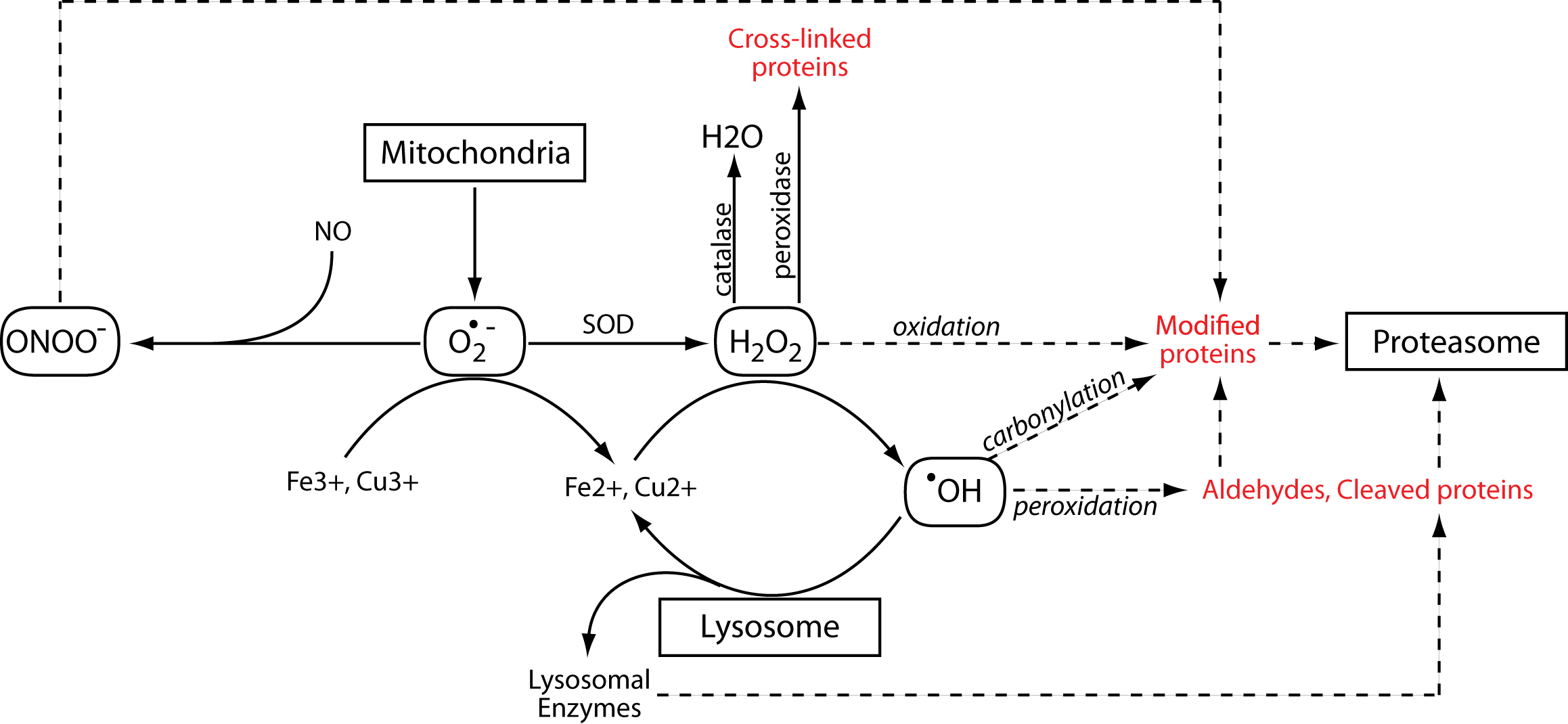

Supplement: Figure S6 — Schematic representing potential pathways for intracellular protein modification and/or degradation. (TIF) [file pone.0020073.s006.tif]

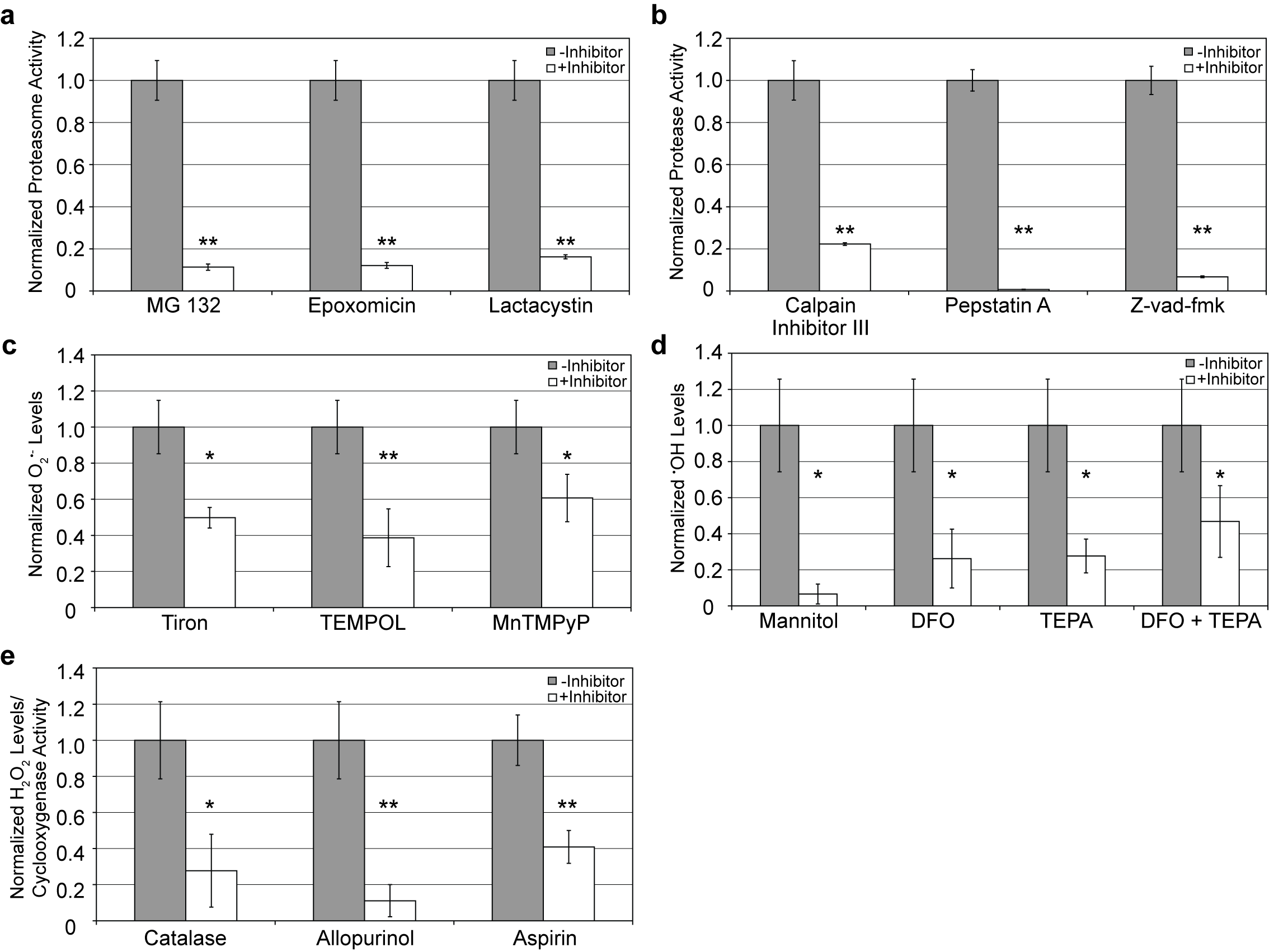

Supplement: Figure S7 — Control assays to validate inhibitor effectiveness. HeLa-fR cells were pretreated with either PBS (gray bars) or indicated inhibitors (white bars) for 1 hour before the addition of 10 µM STS. The PBS/Inhibitors and STS were then incubated with the cells for an additional 24 hours. (a) Proteasome inhibitors were assayed using Proteasome-Glo. (b) Protease inhibitors were assayed using Calpain-Glo (Calpain Inhibitor III), CV-Cathepsin B Detection Kit (Pepstatin A) or Caspase-Glo 3/7 (z-vad-fmk). (c) O2 •- scavengers were assayed using DHE. (d) •OH scavengers were assayed using HPF. (e) H2O2-related scavengers were assayed using CM-H2DCFDA (catalase and allopurinol) or the Cox Activity Assay Kit (aspirin). All measurements were normalized to the mean measurements in the absence of the respective inhibitor. Statistical significance between -inhibitor and +inhibitor is indicated with a * (p<0.05) or ** (p<0.01). (TIF) [file pone.0020073.s007.tif]

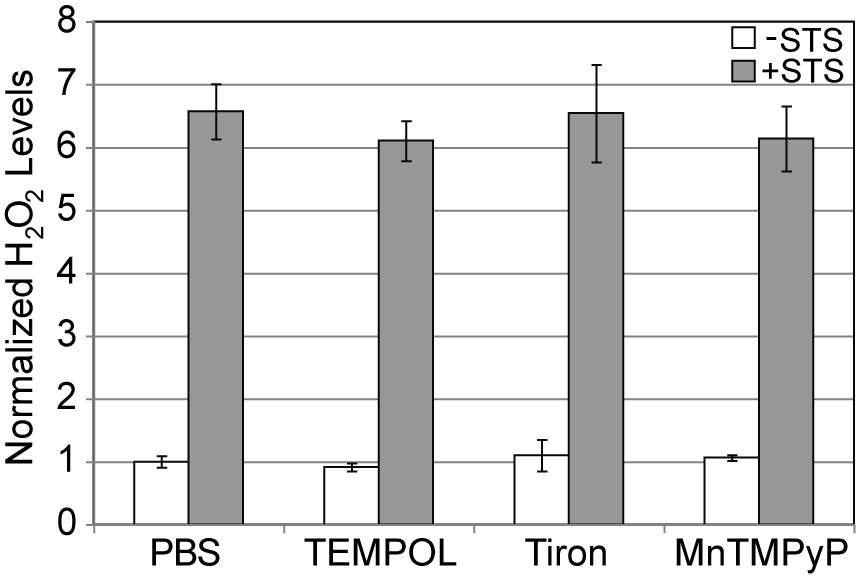

Supplement: Figure S8 — Measurements of H2O2 in HeLa-fR cells pretreated with superoxide scavengers prior to the addition of STS. HeLa-fR cells were pretreated with PBS (control), 10 mM Tiron, 10 mM TEMPOL, or 100 µM MnTMPyp for 1 hour, followed by PBS (white bars) or 10 µM STS (gray bars) for 24 hours. HeLa cells were then assayed for H2O2 levels using CM-H2DCFDA. All measurements were normalized to the mean of PBS-treated controls, in the absence of STS. (TIF) [file pone.0020073.s008.tif]
